# Supplementary material for: A Heterostructure Photoelectrode Based on Two-Dimensional Covalent Organic Framework Film Decorated TiO2 Nanotube Arrays for Enhanced Photoelectrochemical Hydrogen Generation
Source: Molecules. 2023 Jan 13;28(2):822. doi: 10.3390/molecules28020822 (PMC9865276; doi:10.3390/molecules28020822)
Supplement: Supplementary file 1 [file molecules-28-00822-s001.zip › molecules-2157025-supplementary.pdf]

Electronic Supplementary Information for

A Heterostructure Photoelectrode Based on Two-Dimensional Covalent Organic Framework Film Decorated TiO_2 Nanotube Arrays for Enhanced Photoelectrochemical Hydrogen Generation

Yue Zhang, Yujie Li, Jing Yu, Bing Sun*, Hong Shang*

School of Science, China University of Geosciences (Beijing), Beijing 100083, China

* Corresponding author:

B.S., Email: sunbing@cugb.edu.cn;

H.S., Email: shanghong@cugb.edu.cn

S1. Synthesis and characterization of TiO_2 NTAs

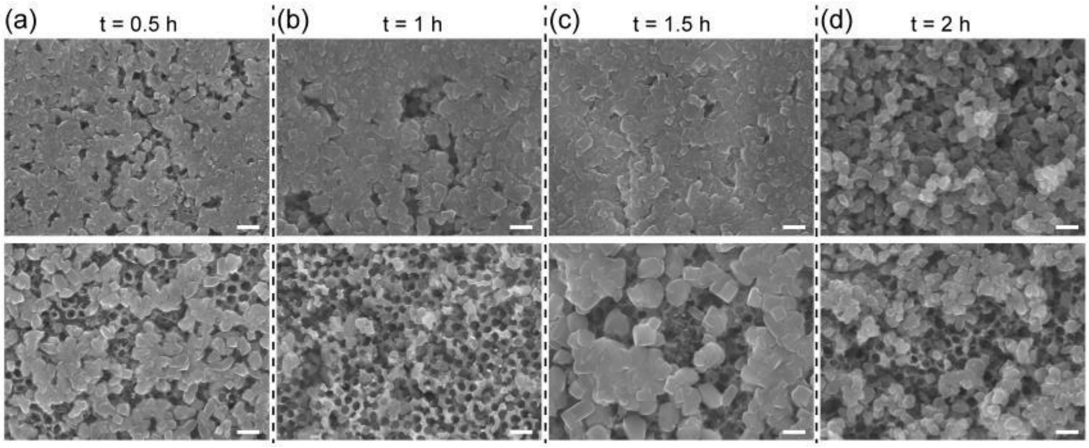

Figure S1. SEM images of TiO_2 nanostructures on Ti foil synthesized by anodization method. Anodization time: (a) 0.5 h, (b) 1 h, (c) 1.5 h, (d) 2 h. The scale bar size is 200 nm for all panels.

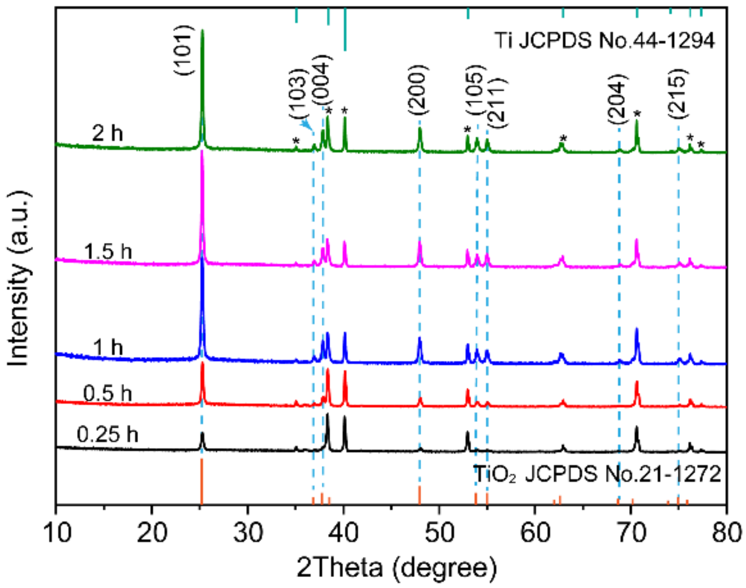

Figure S2. XRD patterns of TiO_2 nanostructures on Ti foil synthesized by anodization method at various reaction times. The * labels represented the typical diffraction peaks of substrate Ti foil.

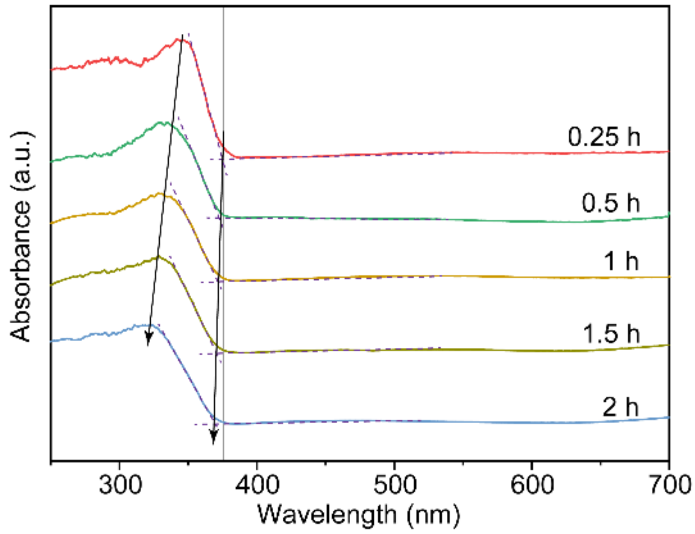

Figure S3. UV-vis diffusive reflection absorption spectra of TiO_2 nanostructures formed on Ti foil at various anodization times.

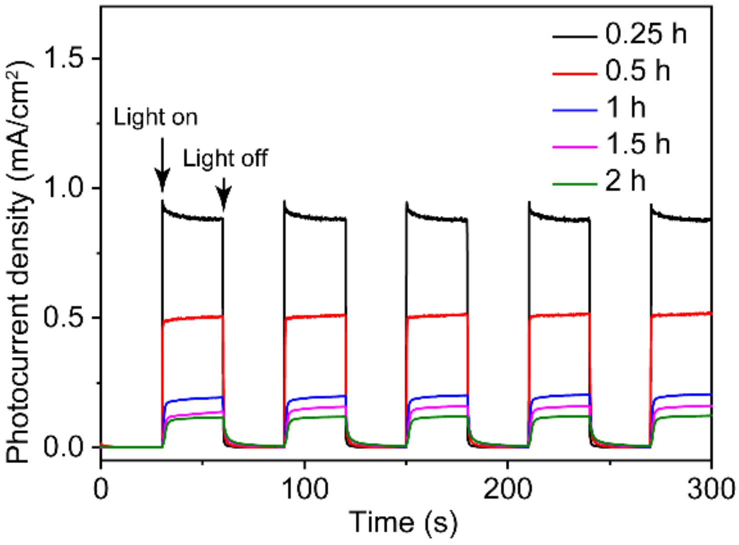

Figure S4. The transient photocurrent response of TiO_2 nanostructures formed on Ti foil at various anodization times. The photocurrent was recorded under full-range light irradiation.

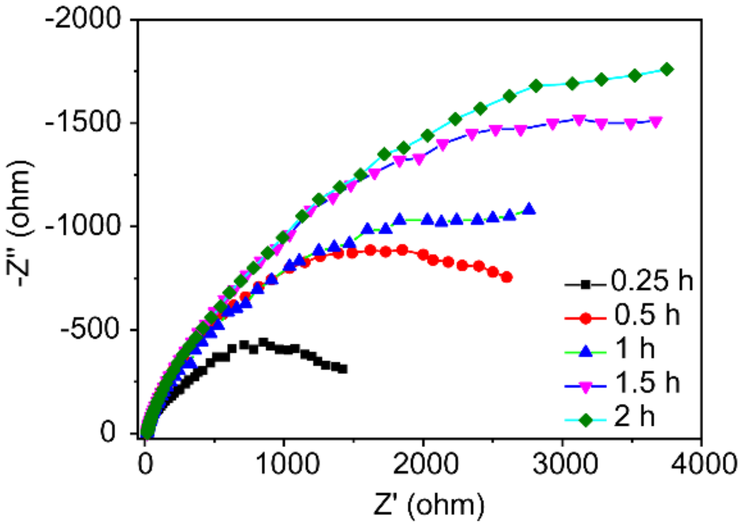

Figure S5. EIS Nyquist plots of TiO_2 nanostructures formed on Ti foil at various anodization times. EIS measurements were performed under full-range light irradiation.

S2. Fabrication of TpPa-1 COF films on TiO_2 NTAs

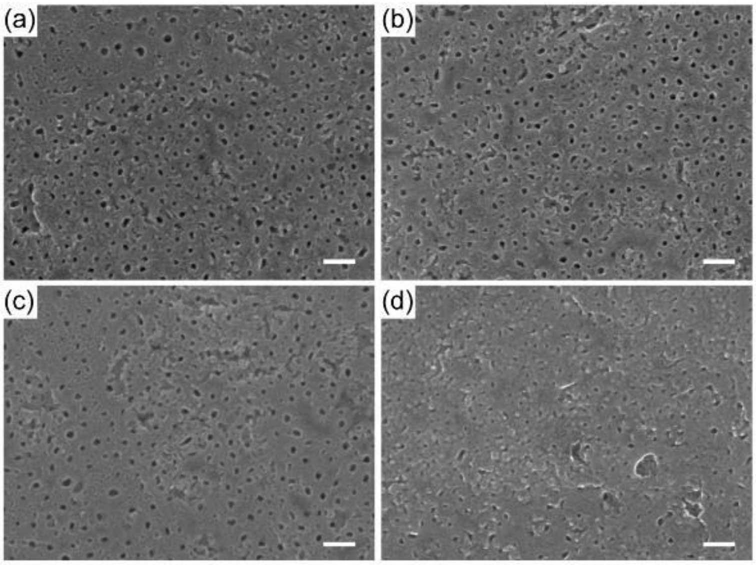

Figure S6. SEM images of TpPa-1/TiO_2-NTAs with (a) 0.4 mM Tp and 0.6 mM Pa, (b) 0.8 mM Tp and 1.2 mM Pa, (c) 1.5 mM Tp and 2.25 mM Pa, and (d) 2 mM Tp and 3 mM Pa. The scale bar size is 200 nm for all panels.

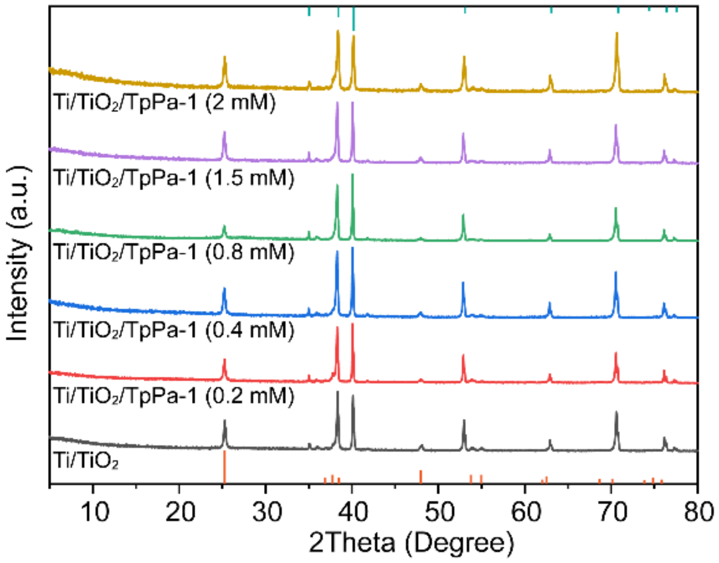

Figure S7. XRD patterns of TpPa-1/TiO_2-NTAs synthesized with different monomer concentrations (based on the concentration of Tp).

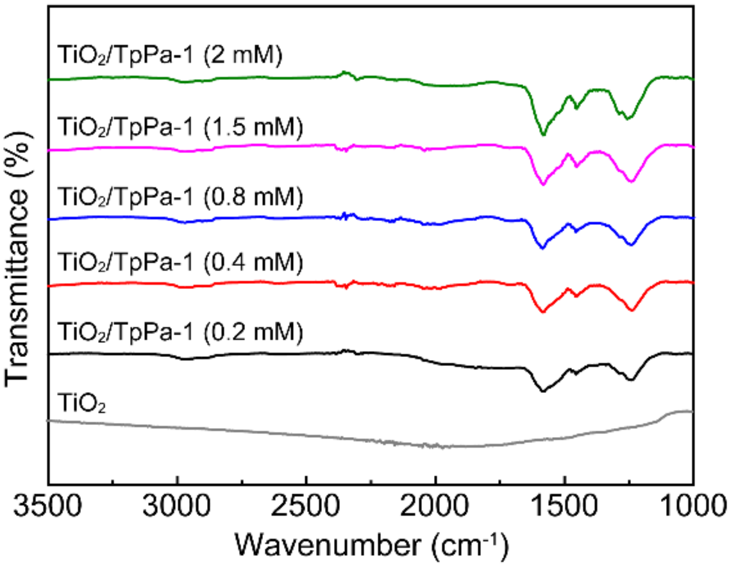

Figure S8. FTIR spectra of Ti/TpPa-1/TiO2-NTAs synthesized with different monomer concentrations (based on the concentration of Tp) compared to TiO_2 NTAs.

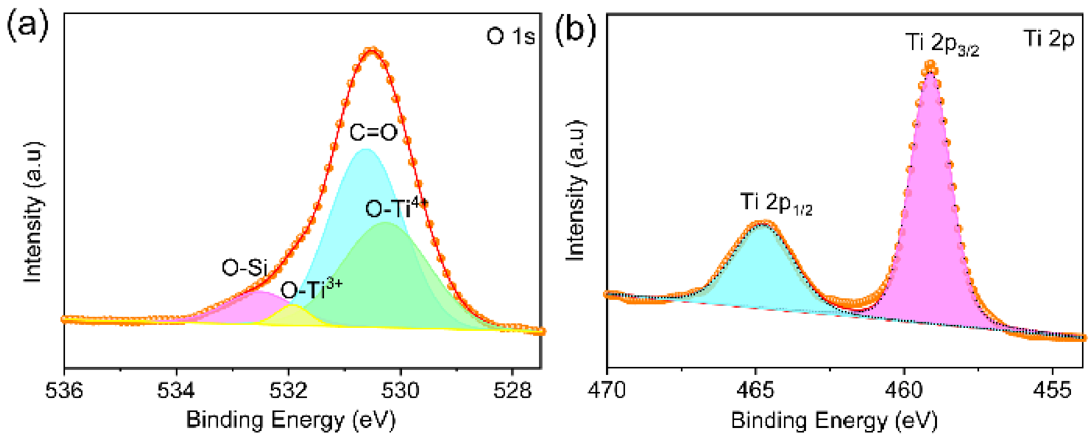

Figure S9. High-resolution XPS spectra of O1s and Ti2p cores of Pt_1@TpPa-1/TiO_2-NTAs.

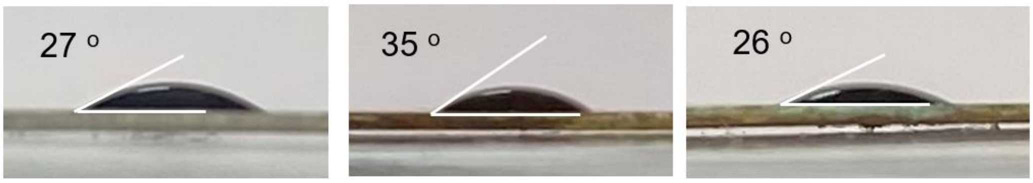

Figure S10. Contact angle measurements of TiO_2 NTAs (left), TpPa-1/TiO_2-NTAs (middle), and Pt_1@TpPa-1/TiO_2-NTAs (right).

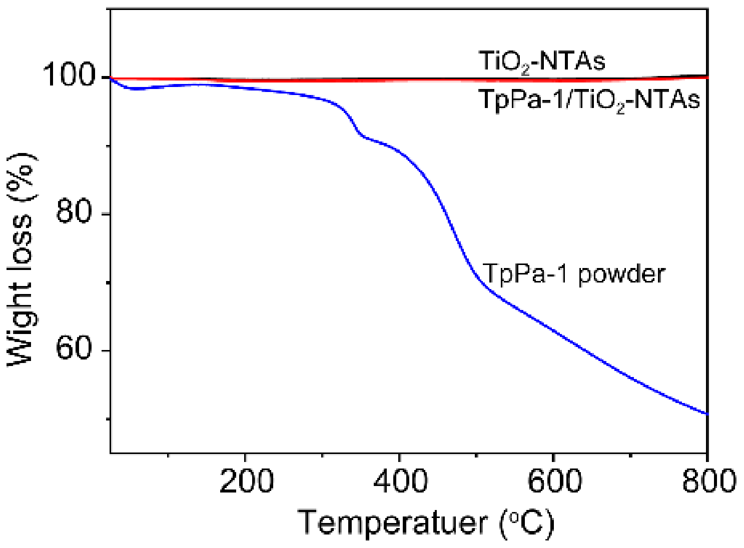

Figure S11. Thermogravimetric analysis (TGA) of TiO_2 NTAs and TpPa-1/TiO_2-NTAs compared to TpPa-1 powders. The TGA measurements were performed on a TechMax EXSTAR 700 Thermogravimetric Analyzer during the temperature range from room temperature to 800 °C with a ramp rate of 5 °C min^{-1} under high-purity nitrogen conditions.

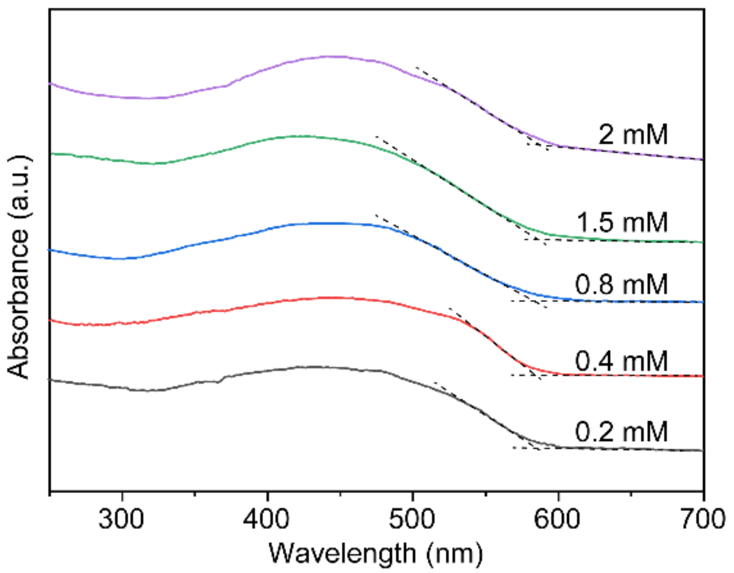

Figure S12. UV-vis diffusive reflection absorption spectra of TpPa-1/TiO_2-NTAs synthesized with different monomer concentrations (based on the concentration of Tp).

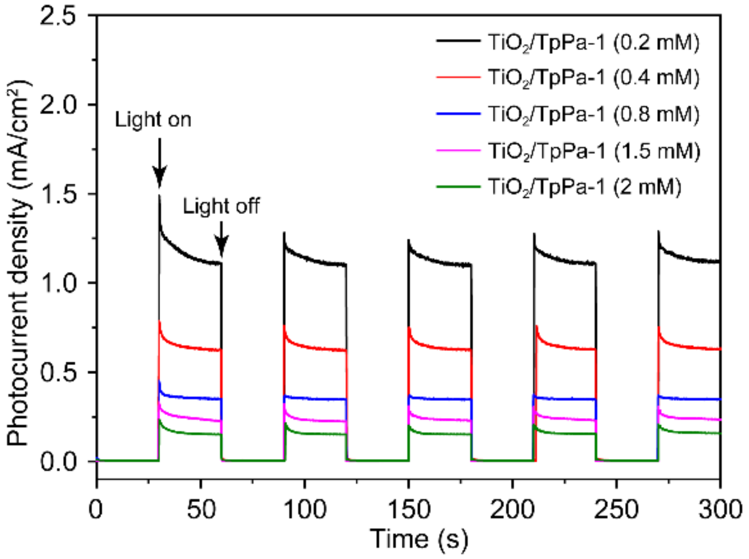

Figure S13. The transient photocurrent response of TpPa-1/TiO_2-NTAs synthesized with different monomer concentrations (based on the concentration of Tp). The photocurrent was recorded under full-range light irradiation.

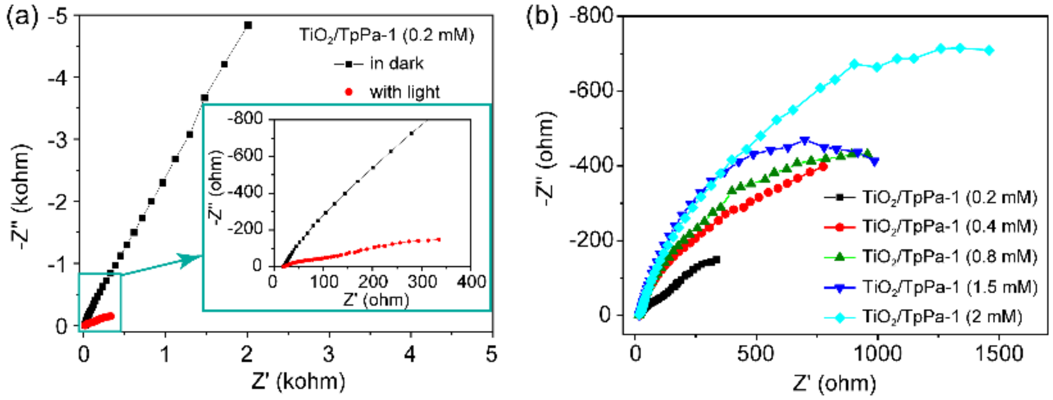

Figure S14. EIS Nyquist plots of (a) TpPa-1/TiO_2-NTAs (0.2 mM) electrodes in dark and with light irradiation, and (b) TpPa-1/TiO_2-NTAs synthesized with different monomer concentrations (based on the concentration of Tp).

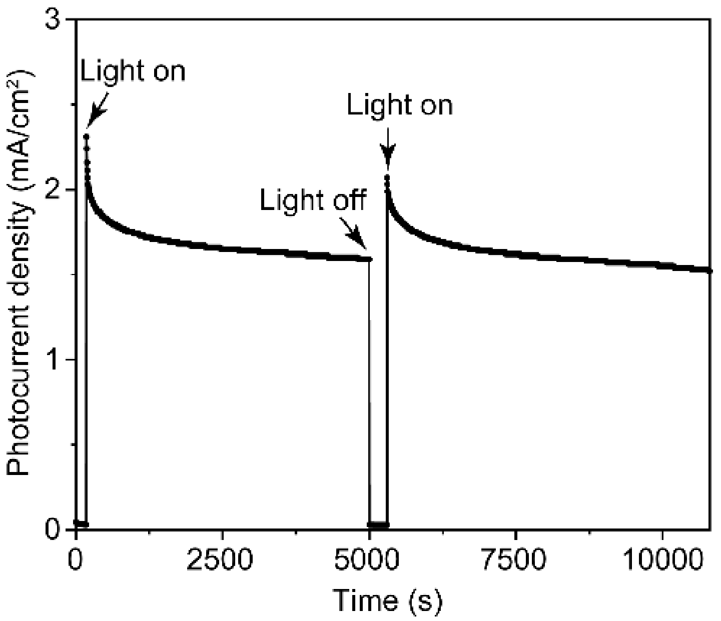

Figure S15. Time-dependent photocurrent density of Pt_1@TpPa-1/TiO_2-NTAs photoelectrode under full-range light irradiation.

S3. Hydrogen evolution performance of Pt_1@TpPa-1/TiO_2-NTAs electrodes.

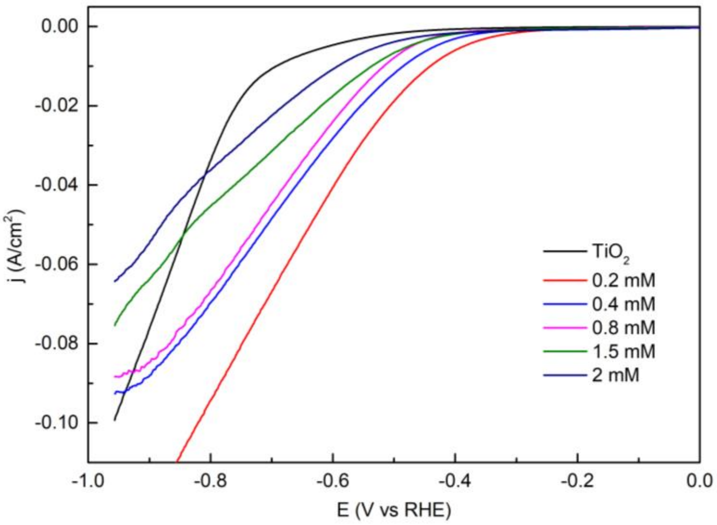

Figure S16. Linear scan voltammetry curves of TpPa-1/TiO_2-NTAs electrode synthesized from different monomer conditions compared to TiO_2 NTAs under full-range light irradiation.

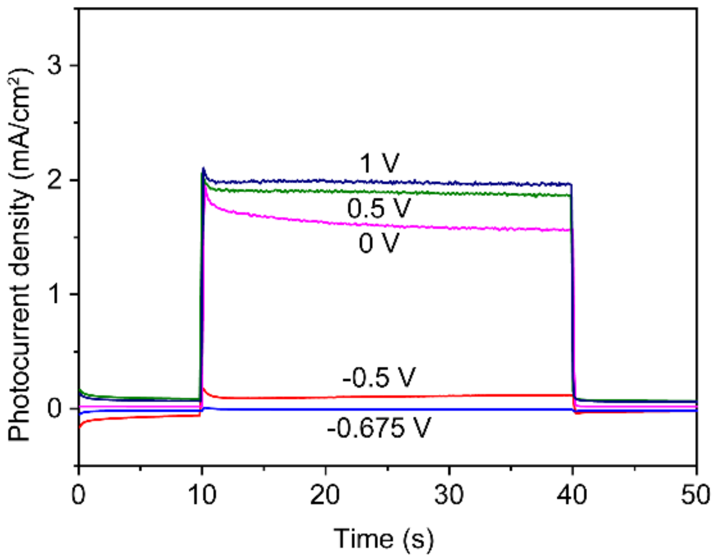

Figure S17. Transient photocurrent response of Pt_1@TpPa-1/TiO_2-NTAs photoelectrode under varying bias voltage vs. SCE.

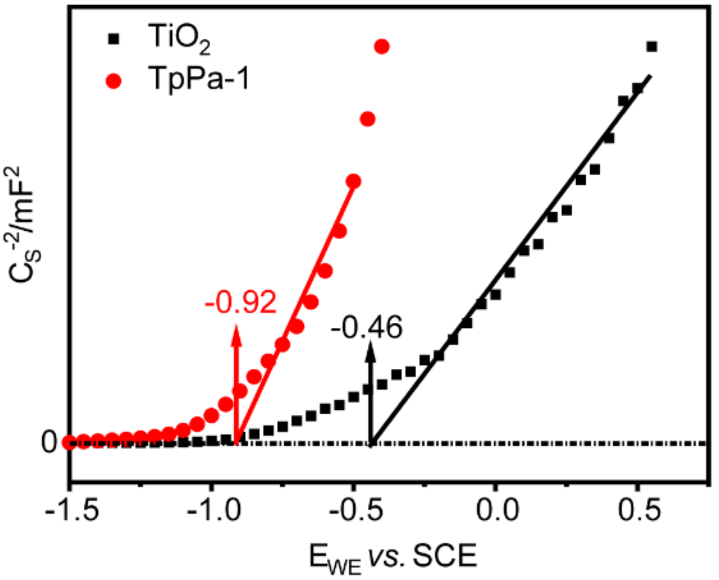

Figure S18. Mott-Schottky plots of TiO_2 and TpPa-1 measured in the dark at a frequency of 1 kHz in 0.5 M Na_2SO_4 aqueous solution.
